# Fatty Acid Composition and Bioactive Profiles in the Aerial Parts of *Cannabis sativa*

**DOI:** 10.3390/molecules30091947

**Published:** 2025-04-27

**Authors:** Weronika Jacuńska, Wioletta Biel, Grzegorz Tokarczyk, Patrycja Biernacka, Grzegorz Bienkiewicz, Katarzyna Janda-Milczarek

**Affiliations:** 1 Department of Monogastric Animal Sciences, Division of Animal Nutrition and Food, West Pomeranian University of Technology in Szczecin, 29 Klemensa Janickiego Street, 71-270 Szczecin, Poland; wioletta.biel@zut.edu.pl; 2 Department of Fish, Plant and Gastronomy Technology, Faculty of Food Sciences and Fisheries, West Pomeranian University of Technology in Szczecin, 4 Papieża Pawła VI Street, 71-459 Szczecin, Poland; grzegorz.tokarczyk@zut.edu.pl (G.T.); patrycja.biernacka@zut.edu.pl (P.B.); 3 Department of Commodity Science, Quality Assessment, Process Engineering and Human Nutrition, Faculty of Food Sciences and Fisheries, West Pomeranian University of Technology in Szczecin, 4 Papieża Pawła VI Street, 71-459 Szczecin, Poland; grzegorz.bienkiewicz@zut.edu.pl; 4 Department of Biology, Parasitology and Pharmaceutical Botany, Faculty of Pharmacy, Medical Biotechnology and Laboratory Medicine, Pomeranian Medical University in Szczecin, 72 Powstańców Wielkopolskich Street, 70-111 Szczecin, Poland

**Keywords:** bioactive compounds, industrial hemp, lipid profile, lipid quality indices, plants, sustainability

## Abstract

The interest in *Cannabis sativa* L. has been on the rise recently, driven by its potential applications in various sectors, including the food industry, the medical sector, and other key areas. This crop possesses a diverse profile of essential fatty acids and a range of bioactive compounds, which exhibit properties that are highly significant for functional food ingredients and nutraceutical purposes. The objective of this study was to investigate the characteristic lipid and bioactive profiles of different plant parts (e.g., inflorescences and leaves) to ascertain their possible uses in nutritional and therapeutic fields. The fat content of the plant material was determined by the Soxhlet method, and gas chromatography was employed for the assessment of the fatty acids and selected bioactive compounds profile. In addition, some lipid quality indices were calculated with the purpose of providing a more in-depth discussion of these aspects beyond the traditional n-6/n-3 ratio. A distinct lipid composition was evident among the various plant parts. Compared to inflorescence samples, leaves typically contain higher proportions of SFAs, MUFAs, PUFAs, and n-3 fatty acids, along with a more favorable n-6/n-3 ratio, which may significantly impact nutritional value. Phytol-rich leaves can suggest its potential application as a functional feed or even a nutraceutical. Furthermore, the occurrence of hexacosane and related antimicrobial and antifungal compounds serves to enhance the practical utility of the leaves. Notably, hemp leaves are not merely a by-product, but rather offer significant practical applications.

## 1. Introduction

The genus *Cannabis* comprises plants with a long history of cultivation, dating back thousands of years. Research indicates that these plants have been cultivated for over 10,000 years, with domestication likely originating in Central Asia [1,2,3,4]. In antiquity, hemp was primarily valued for its durable fibers, which were essential for the production of ropes, textiles, and sails—key materials that contributed to the advancement of maritime navigation and trade [5,6,7]. Additionally, these plants produce a resin containing cannabinoids, which accumulate on glandular trichomes, particularly abundant on female inflorescences [8]. The seeds were used to extract oil but not exclusively; soaking the seeds facilitated the removal of their hulls, yielding a protein-rich liquid that, when concentrated, formed a tofu-like product. Owing to its unique properties, hemp has found applications in both industrial and pharmaceutical sectors. The species *Cannabis sativa* L. is commonly classified and regulated based on its production of psychoactive cannabinoids and has been given various informal names reflecting its diverse commercial and medicinal applications.

The classification of *Cannabis* based on the quantity of bioactive compounds produced includes five chemotypes, designated I to V. Type I plants are characterized by a THCA (tetrahydrocannabinolic acid) to CBDA (cannabidiolic acid) ratio greater than 1 [9,10]. In contrast, type III plants exhibit a lower ratio, below 1, while in chemotype IV, the dominant cannabinoid is CBGA (cannabigerolic acid) [11]. Chemotype V, on the other hand, contains negligible or practically no cannabinoids. From a legal and regulatory standpoint, most countries classify and regulate *Cannabis* based on its delta-9-tetrahydrocannabinol (THC) content. Plants containing less than 0.3% THC are classified as hemp (*C. sativa* subsp. *sativa*) [12], whereas those producing 0.3% or more of THC are categorized as the drug-type variety (*C. sativa* subsp. *indica*). The term “hemp” is generally reserved for varieties cultivated specifically for non-drug applications [12].

In recent decades, the resurgence of interest in hemp, driven by its potential applications in the pharmaceutical, food, and cosmetic industries, has significantly enhanced its relevance. Beyond its commercial value, hemp cultivation offers notable environmental benefits. Its deep root system facilitates soil bioremediation by enabling the uptake and accumulation of pollutants such as cadmium and lead, contributing to soil detoxification and improved land sustainability [13]. Additionally, hemp cultivation requires minimal irrigation, and its late flowering period, occurring at the end of summer, provides habitats for pollinating insects such as bees, thereby supporting biodiversity and enhancing the productivity of other crops [14,15].

Hemp provides many valuable nutrients, primarily fats and proteins. The seeds contain approximately 30–35% fat and 20% high-biological-value protein [16,17]. The fat profile consists mainly of polyunsaturated fatty acids (PUFAs), such as linoleic acid (C18:2 n-6), γ-linolenic acid (C18:3 n-6), and α-linolenic acid (C18:3 n-3). Among the monounsaturated fatty acids (MUFAs), the predominant one is oleic acid (C18:1 n-9) [17,18]. A key aspect is the favorable n-6 to n-3 fatty acid ratio, which can help compensate for n-3 deficiencies in the diet, where saturated fatty acids (SFAs) dominate, increasing the risk of inflammatory and cardiovascular diseases [19,20]. Hemp protein contains all essential amino acids, making it a valuable dietary supplement and a plant-based alternative to animal-derived proteins [18,21,22]. Processed hemp seeds are used in food production, including bread, beverages, bars, and confectionery products [23]. In the food industry, hemp products are primarily associated with oil extracted from seeds, as well as resin-rich oil containing CBD. In agricultural and food processing, the generation of plant-based by-products has become an increasingly important issue because of its environmental impact, particularly concerning the carbon footprint within a sustainable food chain, as well as economic and social considerations. The production of hemp seed oil generates substantial amounts of unused plant material, such as stems and leaves, which remain relatively underutilized and insufficiently studied. Investigating their potential applications is essential for promoting the comprehensive utilization of raw materials and minimizing waste. A notable example of effective plant resource utilization is seen in sugarcane and sugar beets, where both primary and secondary processing products are used across various industries, including biofuel production and animal feed. The processing of plant-based by-products often involves the use of press cakes remaining after juice or seed extraction, which may still contain valuable nutrients and functional compounds [24,25,26]. Companies specializing in this field are increasingly implementing innovative solutions, such as utilizing broccoli leaves and stems or sea buckthorn leaves for the production of herbal infusions [27]. The agricultural sector generates numerous residues that can be repurposed in animal feed. For instance, almond hulls, which are produced in large quantities during almond processing, have been identified as a potential feed ingredient for ruminants because of their beneficial properties [28]. Similarly, olive cake, a by-product of olive oil extraction, can be utilized as an alternative to conventional grains in livestock nutrition, helping to reduce feed costs while simultaneously promoting sustainable agricultural practices by repurposing by-products. This approach not only minimizes waste but also enhances the circular economy by integrating secondary agricultural materials into the feed industry [29,30,31]. Agri-food waste often contains substantial amounts of bioactive compounds, making it a valuable raw material for various industries. In this context, the broader utilization of hemp biomass presents a particularly promising opportunity. Inflorescences, leaves, and stems can be employed not only in the feed industry but also as sources of functional ingredients in animal nutrition. This approach aligns with the principles of a circular economy, contributing to the minimization of plant-based raw material waste and enhancing the sustainability of agricultural and food production systems [32,33].

In the aerial parts of hemp (e.g., leaves, seeds, and inflorescences), more than 500 secondary metabolites have been identified, including over 100 cannabinoids such as CBD and THC. Cannabinoids exhibit anti-inflammatory, antioxidant, analgesic, and anticancer properties [34,35,36]. The anticancer mechanism of action is based on the induction of apoptosis in cancer cells and the modulation of signaling pathways responsible for tumor development [37,38,39]. CBD influences apoptosis through various mechanisms, though its effects on X-linked inhibitor of apoptosis protein (XIAP) expression are complex. XIAP functions as an inhibitor of apoptosis, and its upregulation has been associated with increased resistance to cell death in cancer cells, as demonstrated in previous studies [40]. Therefore, while CBD may modulate apoptosis, the role of XIAP in this process requires careful consideration, as its upregulation could potentially contribute to cancer cell survival rather than promoting apoptosis. Additionally, sesquiterpenes, which are predominantly found in inflorescences, have been shown to reduce the viability of breast cancer cells by interacting with CB2 receptors. These findings suggest a potential role for hemp-derived compounds in cancer therapeutics, particularly in modulating apoptotic pathways and tumor cell viability [41], confirmed in both in vitro and in vivo studies. Terpenes, responsible for the characteristic aroma and flavor of hemp, also exhibit a broad spectrum of biological activity, including antibacterial, anti-inflammatory, and anticancer effects [38]. Flavonoids, such as apigenin, luteolin, kaempferol, and quercetin, further enhance antioxidant, neuroprotective, and anti-inflammatory actions [42,43,44].

In addition to these bioactive compounds, lipids represent another crucial component of hemp’s chemical composition, playing a fundamental role in regulating metabolic processes and overall health. Traditionally, the evaluation of fat quality was based on the ratio of saturated to polyunsaturated fatty acids, as well as the balance between n-6 and n-3 fatty acids. However, recent research suggests that the influence of dietary fats on lipid metabolism and cardiovascular health is far more complex, involving multiple biochemical pathways and interactions that extend beyond simple fatty acid ratios [45,46,47]. Not only total cholesterol levels but also specific fatty acids play a crucial role. Some exhibit atherogenic properties (promoting atherosclerosis), while others have anti-atherogenic effects or influence the risk of thrombosis by either increasing or decreasing the likelihood of clot formation [48]. Considering these differences allows for a more comprehensive assessment of fats in the context of their impact on cardiovascular health, which is particularly important in modern society, where cardiovascular diseases are among the leading causes of death worldwide [49,50]. A crucial step in evaluating lipid quality, beyond identifying their content, is the use of widely applied indices, such as the atherogenic index (AI), thrombogenic index (TI), health-promoting index (HPI), and the hypocholesterolemic/hypercholesterolemic (h/H) ratio [48]. The interpretation of these indices offers a comprehensive assessment of the fatty acid composition and its potential health implications, facilitating the evaluation of overall lipid quality in the diet and their suitability for human consumption. This analysis enhances the understanding of lipid applicability across various sectors, including the development of functional foods, dietary supplements, and cosmetics, while also providing insight into their broader impact on human health.

Research on hemp inflorescences and their derived products primarily focuses on evaluating the antioxidant and antibacterial activity of their extracts [51,52,53,54,55,56]. In the food industry, hemp seeds are the most commonly used component, processed into oils, press cakes, flours, and protein hydrolysates [16,57]. While hemp seeds have been extensively studied for their nutritional value, other parts of the plant, such as leaves, contain unique bioactive compounds that warrant further investigation. Leaves are primarily processed into herbal teas, whereas inflorescences, because of their high concentration of bioactive compounds, serve as a key source for the extraction of high-quality bioactive extracts with potential applications in pharmaceuticals, nutraceuticals, and cosmetics [58,59,60]. Consequently, research has been conducted to evaluate the biological value of fats based on fatty acid composition and quality indices in leaves (H_L) and inflorescences (H_IF) of *Cannabis sativa* subsp. *sativa*.

## 2. Results

The results for dry matter and crude fat content are presented as mean values in Table 1. The levels of these components in the evaluated raw materials varied significantly. Hemp inflorescences (H_IF) contained statistically significantly more dry matter and fat compared to hemp leaves (H_L). The leaves had almost 42% less crude fat than the inflorescences. Descriptive statistics for hemp can be found in Tables S1 and S2 of the Supplementary Materials.

### 2.1. Fatty Acids Profile and Other Substances

**Figure 1:**
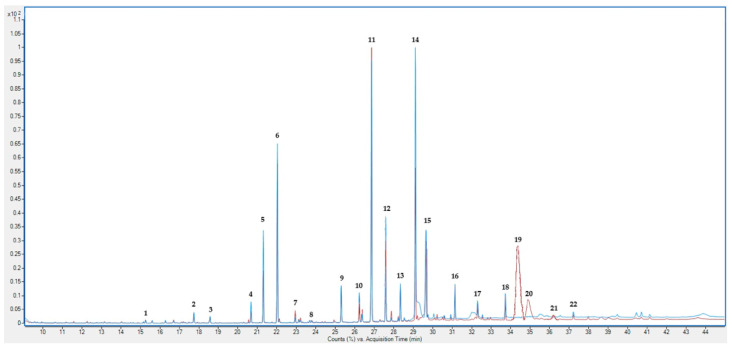
The mass spectra of the detected metabolite in hemp inflorescences (red lines) and leaves (blue lines). The identified compounds correspond to the numbered peaks as follows: (1) lauric acid (C12:0); (2) 2-norpinanol 3,6,6-trimethyl; (3) myristic acid (C14:0); (4) phytyl 2-methylbutanoate; (5) phytol; (6) palmitic acid (C16:0); (7) palmitoleic acid (C16:1); (8) heptadecanoic acid (C17:0); (9) stearic acid (C18:0); (10) oleic acid (C18:1 n-9); (11) C19:0 standard; (12) linoleic acid (C18:2 n-6); (13) eicosanoic acid (C20:0); (14) α-linolenic acid (C18:3 n-3); (15) hexacosane; (16) behenic acid (C22:0); (17) octacosane; (18) lignoceric acid (C24:0); (19) Δ8-tetrahydrocannabinol; (20) cannabidiol; (21) cannabichromene; (22) hexacosanoic acid.

Table 2 presents the fatty acid composition of hemp inflorescences and leaves, expressed as a percentage of the total fat content and converted to % per 100 g of dry matter. The mass spectra of the detected substances in hemp can be found in Figure 1 and Figures S1 and S2 of the Supplementary Materials. Statistically significant differences were observed in the content of all analyzed fatty acids among the examined plant parts. Hemp leaves (H_L) contained higher levels of most saturated fatty acids, including palmitic, stearic, and eicosanoic acids, as well as the dominant polyunsaturated fatty acid, α-linolenic acid (C18:3 n-3). In contrast, inflorescences (H_IF) had a greater proportion of behenic, lignoceric, linoleic, and heptadecanoic acids. A particularly high content of α-linolenic acid was characteristic of both tissues, suggesting their potential as a source of n-3 fatty acids.

A summary of the fatty acid groups and the n-6/n-3 ratio in the hemp inflorescences and leaves, expressed as a percentage of the total fat content, is presented in Table 3. The analysis of the fatty acid compositions in H_IF and H_L revealed statistically significant differences in the content of various fatty acid groups. The main components are polyunsaturated fatty acids (PUFAs), accounting for 34.52% in H_IF and 3.49% in H_L. Monounsaturated fatty acids (MUFAs) were found in higher amounts in H_L (3.28%) compared to H_IF (2.62%). The content of saturated fatty acids (SFAs) was also higher in H_L (35.32%) than in H_IF (3.49%). Similar differences were observed in the total unsaturated fatty acid content, which was 39.77% in H_L and 37.14% in H_IF. Monounsaturated fatty acids (MUFAs) were less abundant, constituting 2.62% in H_IF and 3.28% in H_L. However, saturated fatty acids (SFAs) were present in higher amounts than MUFAs, reaching 26.48% in H_IF and 35.32% in H_L. Among SFAs, the dominant fatty acids were C16:0 (palmitic acid) and C18:0 (stearic acid), which together made up the majority of this fraction. Regarding n-3 fatty acids, their proportion was higher in H_L (29.48%) than in H_IF (26.39%). In contrast, the content of n-6 fatty acids was higher in H_IF (8.13%) compared to H_L (7.00%). The n-6/n-3 ratio indicated more favorable proportions in H_L (0.24) than in H_IF (0.31).

The contents of other lipid substances in the hemp inflorescences and leaves are presented in Table 4. A significantly higher concentration of compounds such as 2-norpinanol 3,6,6-trimethyl (1.55% in H_IF vs. 0.48% in H_L) and phytyl, 2-methylbuanoate (2.92% in H_IF vs. 0.65% in H_L) was observed in H_IF. Phytol was dominant in H_L (9.55% vs. 3.90% in H_IF). For hexacosane, a higher content was found in H_L (12.21% vs. 10.33% in H_IF), as well as for octacosane (1.69% in H_L vs. 1.19% in H_IF). Cannabinoids such as cannabidiol (CBD) (2.03%), cannabichromene (CBC) (5.78%), and Δ8-tetrahydrocannabinol (Δ8-THC) (7.83%) were detected in H_IF and not detected in H_L. Because of the fact that the method is not suited for quantitative and qualitative determinations of cannabinoids, the potential bias in the relative composition due to differences in the mass detector response across chemical classes may occur. The results represent a compositional profile rather than absolute quantification. While the findings are informative, a dedicated cannabinoid extraction and standardization approach would be needed for precise quantification. The compound hexacosanoic acid, methyl ester was present in both plant parts but in higher concentrations in H_IF (0.86%) compared with H_L (0.34%).

### 2.2. Lipid Quality Indices

All lipid quality index values were significantly different among the examined plant parts (Table 5). The atherogenic index (AI), which measures the potential risk of atherosclerosis, was higher in H_L (0.58) than in H_IF (0.39). The modified AI index, which additionally considers C18:0 acid, showed a value of 4.66 in H_L and 3.02 in H_IF.

The thrombogenic index (TI), which relates to the risk of blood clot formation, was also higher in H_L (0.24) compared to H_IF (0.17).

Indices associated with hypocholesterolemic and health-promoting effects showed more favorable differences for H_IF. The h/H ratio (hypocholesterolemic to hypercholesterolemic fatty acids) was higher in H_IF (2.94) compared to H_L (1.91). The health-promoting index (HPI) also reached higher values in H_IF (2.58) than in H_L (1.73).

## 3. Discussion

The chemical composition of hemp is shaped by a complex interplay of genetic and environmental factors. Genetic variability among different cultivars significantly affects the presence and concentration of various bioactive compounds, including cannabinoids, terpenes, and phenolic compounds. These distinct chemical profiles can enhance the suitability of specific plant parts for targeted industrial applications. The highest concentration of bioactive compounds is found in female hemp inflorescences, which possess a dense covering of glandular trichomes—specialized structures where these metabolites are synthesized and secreted. This makes inflorescences the primary source of cannabinoids and other valuable phytochemicals [8]. This is the primary factor determining their usefulness. Leaves, in contrast, appear to be a less valuable part of the plant and are often considered a by-product, mainly used for composting [61]. The chemical composition of the aerial parts of the plant can vary depending on several factors, such as variety, growth stage at harvest, and environmental conditions [56,62,63]. Hemp leaves and inflorescences contain lower fat content, and their lipid profile differs from that of seeds. In hemp seed oil, the dominant fatty acids include linoleic acid (C18:2n-6) and α-linolenic acid (C18:3n-3), with PUFA levels reaching approximately 79–81% and a favorable n-6/n-3 ratio around 3:1, as confirmed in both Da Porto et al. [64] and Razmaitė et al. [16]. In contrast, our study showed that inflorescences and leaves contain much lower PUFA levels (34.5% and 3.5%, respectively) and substantially higher SFA content. Despite lower total unsaturated fat, the n-6/n-3 ratio was more favorable in leaves (0.24), potentially offering specific metabolic advantages. In inflorescences, the dominant fatty acids among the total fatty acid content include α-linolenic acid, linoleic acid, palmitic acid, oleic acid, and stearic acid [65].

The obtained results allow for an in-depth analysis of the chemical composition of the inflorescences and leaves of *Cannabis sativa* subsp. *sativa*, as well as their potential applications in various fields, such as food, feed, and the pharmaceutical industry. The fatty acid profile in the examined plant parts showed significant differences that may have important practical implications. In the inflorescences, the predominant fatty acids in terms of percentage were C16:0, C18:3 n-3, and C18:2 n-6, while in the leaves, C18:3 n-3 and C16:0 were the most abundant. Studies by other authors confirm that these fatty acids may be present in the highest proportions in the inflorescences [65]. The observed content of α-linolenic acid is consistent with literature reports, indicating that the lipid composition of hemp could serve as a potential source of n-3 fatty acids for the food and pharmaceutical industries [66]. The significant presence of saturated fatty acids, mainly palmitic acid, may raise concerns regarding its impact on health [20]. However, studies suggest that the presence of PUFAs and MUFAs in such proportions may counteract the negative effects of excessive SFAs on blood lipid parameters [67]. Therefore, the content of these components should be interpreted in the context of the total fatty acid intake of the diet.

Unsaturated fatty acids dominated in both the analyzed inflorescences and leaves, aligning with the characteristic chemical profile of hemp. The n-6/n-3 ratio, which reflects the proportion of these fatty acid groups, was more favorable in leaves than in inflorescences, indicating a higher prevalence of health-promoting n-3 fatty acids in the leaves. These proportions significantly exceed the recommended minimum, suggesting the potential use of these extracts in products promoting cardiovascular and nervous system health [68]. The leaves exhibited a higher proportion of PUFAs and a lower n-6/n-3 ratio compared to the inflorescences, suggesting their potential as an alternative raw material for dietary purposes. The inflorescences, in contrast, exhibited more favorable values for the h/H ratio (hypocholesterolemic to hypercholesterolemic fatty acids) and the health-promoting index (HPI), suggesting a greater potential for reducing the risk of cardiovascular diseases compared to leaves. Conversely, the analyzed leaves showed higher values for the atherogenic index (AI) and thrombogenic index (TI), which may indicate a lower overall health-promoting potential in terms of lipid metabolism. In this study, the lipid quality indices were compared with the fatty acid composition of hemp inflorescences as reported by Piovesana et al. [65], providing further context for evaluating the nutritional and functional properties of different plant parts. The AI value, calculated based on their data, ranged from 0.22 (for the Felina 32 variety) to 0.48 (for the Fedora 17 variety). In our study, the AI index was 0.39 for inflorescences and 0.58 for leaves. For the TI index, which based on Piovesana’s data ranged from 0.09 (Futura 75 variety) to 0.24 (Kompolti variety) [65]. For the inflorescences analyzed in our study, the TI index value was 0.17, while for the leaves, it was 0.24. The ratio of hypocholesterolemic to hypercholesterolemic fatty acids (h/H) in the varieties described in Piovesana’s study ranged from 2.43 (for the Finola variety) to 4.68 (for the Felina 32 variety) [65]. In our study, the h/H value was 2.94 for inflorescences and 1.91 for leaves. Similarly, the HPI, which was highest for the Felina 32 variety (4.55) and lowest in the Fedora 17 inflorescences (2.10), according to Piovesana’s data, was found to be 2.58 for inflorescences and 1.73 for leaves in our study. Compared to the inflorescences and leaves analyzed in our study, hemp seed oil shows markedly more favorable lipid quality indices. According to Siol et al. [69], its AI and TI values were substantially lower (0.07 and 0.11, respectively), while both the HPI and h/H were higher (15.35 and 15.55), indicating a much greater potential for supporting cardiovascular health. These findings highlight the nutritional value of hemp seed oil as a rich source of beneficial fatty acids, clearly surpassing the health-promoting potential of the lipid fractions found in vegetative parts such as inflorescences and leaves [69].

Compounds such as phytol, hexacosane, cannabidiol (CBD), cannabichromene (CBC), and Δ8-tetrahydrocannabinol (Δ8-THC) differentiate inflorescences and leaves in terms of their functional and bioactive potential. Phytol is a degradation product of chlorophyll in plants that provides protection against oxidative stress. Because of these properties, phytol and its metabolic products have therapeutic applications. In animal diets, it is used as a dietary supplement with antioxidant properties, supporting the reduction in cholesterol, cortisol, and glucose levels [70,71,72,73]. It is present in the aerial parts of the plant, as well as in the seeds, though in limited amounts. A higher level of phytol was observed in hemp leaves compared to inflorescences, both in this study and as reported by other authors [74].

Hexacosane exhibits strong antibacterial activity against *Klebsiella pneumoniae*, *Salmonella typhi*, *Staphylococcus aureus*, and *Proteus vulgaris*, as well as antifungal and antioxidant properties [75]. Its presence in hemp may indicate its antibacterial and antifungal potential. For comparison, in methanolic extracts of *Citrus limon* leaves, hexacosane was detected as a prominent component, with a GC/MS peak intensity of 21% [76].

Cannabinoids are key bioactive compounds present in hemp, occurring in both industrial and psychoactive varieties. They interact with the endocannabinoid system and have been shown to exert therapeutic effects in various conditions, including anxiety, pain, and inflammation [35,38,77,78,79]. The highest concentrations of CBD are found in the glandular trichomes of hemp inflorescences, where its biosynthesis and secretion occur [80,81,82]. The CBD content in hemp inflorescences can vary significantly depending on the species, cultivation practices, and environmental conditions. Studies indicate that the average CBD concentration in inflorescences is approximately 0.34% [83]. However, hemp cultivated for medical purposes can reach CBD levels as high as 10%, though such high concentrations may lead to exceeding the legal THC threshold of 0.3% [62,63]. In the analyzed inflorescences, the CBD content was 2.03%, while no detectable levels were found in the leaves. After converting the percentage content in total fatty acids and other lipid substances to its concentration per 100 g of dry matter, the CBD level in inflorescences was determined to be 0.26%.

Another cannabinoid present in hemp is cannabichromene (CBC), which undergoes degradation as the plant matures. CBC influences transient receptor potential (TRP) channels, particularly TRPA1, which are involved in pain perception and inflammatory responses [84,85]. This suggests that CBC may be useful for pain relief and the modulation of gastrointestinal motility, further expanding its therapeutic potential [85]. For example, studies have shown that CBC can inhibit excessive gastrointestinal motility induced by inflammation, highlighting its potential role in alleviating conditions such as irritable bowel syndrome [86]. The values obtained in our study for inflorescences were 5.78% in total fatty acids and other lipid substances, which, after conversion, corresponded to 0.73% per 100 g of dry matter Previous research has indicated that the highest CBC concentrations occur around the fourth week of glandular trichome development, after which the levels begin to decline [87]. The concentration range, depending on the cultivation method—whether greenhouse or open-field—can vary from 0.64% to 1.12% [87].

Δ8-Tetrahydrocannabinol (Δ8-THC) is an isomer of the more well-known Δ9-tetrahydrocannabinol/dronabinol (Δ9-THC), the primary psychoactive component of hemp [88]. Δ8-THC has a chemical structure similar to Δ9-THC, with the only difference being the position of the double bond within the carbon chain, which affects its pharmacological properties. Although Δ8-THC is not explicitly regulated in many jurisdictions, it has been shown to produce psychotropic effects similar to those of Δ9-THC, albeit with an estimated potency that is 2–3 times lower [89,90]. Δ8-THC was detected in the inflorescences but not in the leaves, with a concentration of 7.83% in total fatty acids and other lipid substances, corresponding to 0.99% per 100 g of dry matter. The presence of trace amounts of Δ9-THC (<0.3%) in industrial hemp does not produce psychoactive effects, making the plant safe for agricultural production and for use in food and medical applications. However, Δ9-THC was not detected in the analyzed plant parts. It is primarily found in the upper portions of the plant, where trichomes are concentrated, although seeds may also contain small amounts [91]. The challenge in distinguishing these two isomers arises from their nearly identical mass fragmentation patterns in GC-MS analysis. Both Δ8-THC and Δ9-THC exhibit similar fragmentation pathways, leading to overlapping ion peaks that make unambiguous identification difficult. Literature reports indicate that even under optimized conditions, complete separation of Δ8-THC and Δ9-THC often requires additional analytical approaches such as liquid chromatography coupled with mass spectrometry (LC-MS/MS) using reference standards [92].

Hexacosanoic acid may be undetectable or present in low amounts in both inflorescences and leaves, as confirmed by previous studies [74]. In the material analyzed in this study, its concentration was 0.86% (0.11% per 100 g DM) in inflorescences and 0.34% (0.02% per 100 g DM) in leaves.

The growing interest in hemp inflorescences is largely driven by their diverse and abundant bioactive compound profile. Their intricate structure, characterized by a high density of glandular trichomes, offers an extensive surface area for the synthesis and accumulation of cannabinoids and terpenes, further increasing their commercial value and potential applications in various industries [93,94]. In contrast, leaves, while they may contain certain cannabinoids, do not exhibit the same concentration or broad diversity of these compounds as inflorescences, resulting in a lower perceived value [95,96].

The presence of bioactive compounds, such as cannabidiol (CBD) and Δ8-tetrahydrocannabinol (Δ8-THC), suggests potential interactions between lipid components and phytocannabinoids. The mass spectra analysis of hemp inflorescences (red lines) and leaves (blue lines) revealed distinct metabolic profiles, with 22 identified compounds (Figure 1). Inflorescences exhibit a higher concentration of phytocannabinoids (2.03% and 7.83%, respectively), making them a valuable source of bioactive compounds used in medicine and pharmacology. These results highlight the significance of tissue selection in phytochemical investigations and are consistent with earlier data on the variable metabolite distribution in *Cannabis sativa* [97]. Studies suggest that these compounds may modulate the activity of the endocannabinoid system, which could be significant for the treatment of inflammatory and neurodegenerative diseases. The application of Δ8-THC, a less psychotropic isomer of Δ9-THC, may open new therapeutic opportunities, particularly in cases requiring modulation of the endocannabinoid system without pronounced psycho-active effects [98,99]. Leaves, on the other hand, because of their high contents of octacosane, hexacosane, and α-linolenic acid, may play a crucial role in plant defense mechanisms and potential applications in cosmetology and dietary supplements [100]. Octacosane, a long-chain alkane, is one of the dominant compounds in this group, accounting for 11.60% in inflorescences and as much as 18.95% in leaves, while the hexacosane content is 0.83% in inflorescences and 0.90% in leaves.

## 4. Materials and Methods

### 4.1. Plant Material

**Figure 2:**
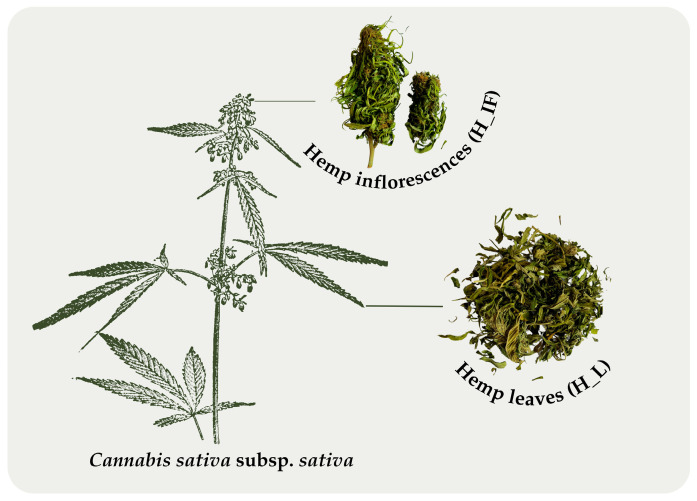
Plant material used for analysis.

The research material consisted of hemp (*Cannabis sativa* subsp. *sativa*) (Figure 2) cultivar Futura, harvested in August 2023. The plants were sourced from a certified organic farm located in Wiekowice, Poland (54°17′59″ N 16°21′39″ E). Sowing was conducted in April of the same year.

The harvest was conducted manually using tools such as pruning shears to minimize mechanical damage to the plant material. Plants were cut at the basal end, approximately 20 cm above the soil surface, then separated into inflorescences and leaves, weighed, and dried at room temperature (18–21 °C) for 3–4 days. Until analysis, the dried plant material was stored in airtight plastic bags, protected from light, at room temperature. The study focused on plant parts classified as inflorescences (H_IF) and leaves (H_L). Before analysis, the material was ground using a KNIFETEC 1095 laboratory mill (Foss Tecator, Höganäs, Sweden).

### 4.2. Chemical Composition

Dry matter

The dry matter (DM) content was determined by drying the plant material at 105 °C until a constant weight was achieved, following the AOAC method 945.15 [101]. After measuring the water content, the dry matter was calculated using the following Equation (1):

DM = 100 − % moisture(1)

Crude fat

The content of crude fat (CF) in the plant material was measured using the Soxhlet extraction method with diethyl ether (method 920.39) [101].

### 4.3. Fatty Acids Profile and Cannabinoids

Chloroform–methanol extracts for fatty acids and cannabinoids determination were prepared using the Bligh and Dyer method [102]. The Bligh and Dyer method was primarily chosen for lipid extraction and not optimized for cannabinoid extraction. Cannabinoids detected in the chloroform phase represent only a fraction of their total content. More targeted cannabinoid extraction method would likely yield different proportions.

The extract was prepared using 5 g of dried and crushed inflorescences and leaves of hemp and a 1:1 mixture of chloroform and methanol. The extracts were filtered and stored in dark bottles. They were then used to prepare fatty acid methyl esters (FAMEs) according to the AOAC method [103]. Cannabinoids were determined together with fatty acid methyl esters. A total of 2 mL of extract was added to 30 mL volume flasks, and the solvent was evaporated under vacuum conditions to obtain 20 mg of fat. The sample obtained in this way was saponified by adding 1 mL of 0.5 M methanolic sodium hydroxide solution. The sample was heated in a heating mantle at 75 °C for 10 min. Then, 5 mL of triboronfluoride in methanol was added and heated for another 15 min to obtain fatty acid methyl esters. A total of 1 mL of n-hexane was added to the sample and shaken for 30 s. The hexane layer contains FAMEs, and cannabinoids were collected, using a Pasteur pipette, in a 2 mL chromatographic vial. The fatty acid composition and cannabinoids of herbal raw materials were studied using gas chromatography coupled to mass spectrometry (GCMS). Chromatographic separations were performed on a gas chromatograph coupled to mass spectrometry 7890A/5975C (Agilent Technologies, Santa Clara, CA, USA) using a HP-5 ms capillary column (100 m × 0.25 mm × 0.25 μm, Agilent Technologies, USA). The injector temperature was 240 °C. The separation was performed in the temperature gradient mode from 140 to 240 °C at a speed of 4 degrees/min. The injection volume was 1 μL, and the analysis time was 45 min. Detection was performed in the SCAN mode in the range 38–400 m/z. The carrier gas flow through the column was 1.0 mL/min [104]. Identification of fatty acid methyl esters was performed by comparing the retention time (tR) of the sample fatty acids with the retention times of the Supelco FAME reference mixture and using the NIST 05 and WILEY 2007 mass spectral libraries. Cannabinoids were identified by a comparison of the mass spectra using the NIST 05 mass spectral library. The identified fatty acids and cannabinoids were calculated as their percentage share in fat. The percentage share of individual fatty acids and cannabinoids was calculated, considering the sum of the integrated fatty acids and cannabinoids areas as 100% and the integrated area of individual fatty acids and cannabinoids as X%.

### 4.4. Lipid Quality Indices

The indices used to assess lipid quality serve as tools for evaluating the nutritional value of fats and oils in the diet, particularly in relation to their potential impact on health (Table 6). Indices such as the atherogenic index (AI) and thrombogenic index (TI) provide broader insight into the fatty acid composition and its potential implications for cardiovascular health [48]. AI is calculated based on the ratio of specific fatty acids that influence atherogenesis, while TI assesses the potential of lipids to induce thrombosis [105,106]. Lower values of these indices indicate more favorable lipid profiles, which may contribute to a reduced risk of ischemic heart disease. The h/H ratio evaluates the balance between cholesterol-lowering fatty acids (hypocholesterolemic fatty acids—DFA) and those that may increase cholesterol levels (hypercholesterolemic fatty acids—OFA) [48]. A higher h/H ratio suggests a greater proportion of beneficial fatty acids, which is associated with a reduced risk of cardiovascular diseases. The health-promoting index (HPI), which is the inverse of the AI, indicates a greater potential for positive health effects when its value is higher [107].

### 4.5. Statistical Analyses

Analyses of the parameters tested were performed in triplicate. Statistical analysis of the obtained results was performed using the Statistica 13 program. The Shapiro–Wilk test was used to check whether the distribution of the obtained results followed a normal distribution. The distribution of the data obtained during the analyses differed from the normal distribution, so the non-parametric Kruskal–Wallis test was used to assess the significance of differences among the parameters studied. In the tables, the results are presented as the mean values and standard deviations, while medians and quartile ranges were used for statistical analyses. Differences were considered statistically significant at *p* ≤ 0.05.

## 5. Conclusions

The study revealed variations in the biological value of lipids extracted from hemp inflorescences and leaves. Chemical analysis showed that hemp leaves contain higher levels of unsaturated fatty acids, including polyunsaturated fatty acids. However, because of the overall higher fat content in inflorescences (12.63% in inflorescences vs. 7.35% in leaves), they serve as a better source of polyunsaturated fatty acids. The n-6/n-3 ratio was found to be more favorable in leaves (0.24) compared to inflorescences (0.31), indicating their superior nutritional and health potential. The health-promoting index (HPI) was significantly higher in inflorescences, whereas the atherogenic (AI) and thrombogenic (TI) indices were notably elevated in leaves. This suggests differing impacts of these plant parts on lipid metabolism in the body. Beyond fatty acid profiles, inflorescences are the primary source of cannabinoids, particularly cannabidiol, which exhibits antioxidant and antibacterial properties. The CBD content in inflorescences was 0.26%, while it was not detected in leaves. However, leaves contain higher percentages of other bioactive compounds like phytol, hexacosane, and octacosane, which may contribute to their functional and nutritional applications.

Inflorescences are richer in fats and bioactive compounds, whereas leaves provide a more balanced profile of unsaturated fatty acids along with additional health benefits. While inflorescences are widely valued for their high contents of bioactive compounds, research findings indicate that leaves—often regarded as a by-product—may serve as a valuable and cost-effective raw material with broad application potential. Future research should focus on comparing different hemp varieties, as genetic variability may lead to significant differences in fatty acid composition, cannabinoid content, and other bioactive compounds. Moreover, cultivation conditions, including organic versus conventional agricultural systems, may influence the chemical composition of the raw material. The utilization of plant-based by-products is a crucial aspect of sustainable production and circular economy principles. For hemp leaves, determining optimal applications is essential to maximize their nutritional and functional potential.

## Figures and Tables

**Table 1:** Dry matter (g/100 g) and crude fat (g/100 g DM) contents in the inflorescences (H_IF) and leaves (H_L) of hemp.

Item	H_IF	H_L
	Mean (±SD)	
Dry matter (DM) [g/100 g]	89.68 (±0.05) ^a^	89.11 (±0.03) ^b^
Crude fat (CF) [g/100 g DM]	12.63 (±0.14) ^a^	7.35 (±0.22) ^b^

a, b—Values in the same row with different superscript letters are significantly different at *p* ≤ 0.05. SD—Standard deviation (*n* = 3).

**Table 2:** Only fatty acids (% of DM) content in the inflorescences (H_IF) and leaves (H_L) of hemp in chloroform extract mixture obtained by the Bligh and Dyer method.

Fatty Acid	H_IF	H_L
	% *	
	Mean (±SD)	
C12:0	0.27 ^a^ (±0.05)	0.31 ^b^ (±0.00)
C14:0	0.63 ^a^ (±0.03)	0.92 ^b^ (±0.01)
C16:0	11.60 ^a^ (±0.31)	18.95 ^b^ (±0.42)
C17:0	0.74 ^a^ (±0.02)	0.23 ^b^ (±0.00)
C18:0	2.63 ^a^ (±0.08)	4.08 ^b^ (±0.12)
C20:0	2.53 ^a^ (±0.06)	4.03 ^b^ (±0.22)
C22:0	4.80 ^a^ (±0.40)	3.79 ^b^ (±0.03)
C24:0	3.27 ^a^ (±0.19)	3.00 ^b^ (±0.18)
C16:1 n-7	0.39 ^a^ (±0.01)	1.29 ^b^ (±0.01)
C18:1 n-9	2.23 ^a^ (±0.02)	1.99 ^b^ (±0.11)
C18:2 n-6	8.13 ^a^ (±0.07)	7.00 ^b^ (±0.22)
C18:3 n-3	26.39 ^a^ (±0.53)	29.48 ^b^ (±0.16)

* The percentage content in total fatty acids and other fatty substances determined. a, b—Values in the same row with different superscript letters are significantly different at *p* ≤ 0.05. SD—Standard deviation (*n* = 3).

**Table 3:** Groups of fatty acids and the n-6/n-3 ratio in the inflorescences (H_IF) and leaves (H_L) of hemp in a chloroform extract mixture obtained by the Bligh and Dyer method.

Item	H_IF	H_L
	% *	
	Mean (±SD)	
SFAs	26.48 ^a^ (±0.21)	35.32 ^b^ (±0.24)
MUFAs	2.62 ^a^ (±0.54)	3.28 ^b^ (±0.10)
PUFAs	34.52 ^a^ (±0.54)	36.49 ^b^ (±0.18)
UFAs	37.14 ^a^ (±0.53)	39.77 ^b^ (±0.25)
n-3	26.39 ^a^ (±0.53)	29.48 ^b^ (±0.16)
n-6	8.13 ^a^ (±0.07)	7.00 ^b^ (±0.22)
n-6/n-3	0.31 ^a^ (±0.01)	0.24 ^b^ (±0.01)
Σ FAs	63.62	75.09
Σ OS	36.38	24.91

* The percentage content in total fatty acids and other fatty substances determined. a, b—Values in the same row with different superscript letters are significantly different at *p* ≤ 0.05. SD—Standard deviation (*n* = 3); FAs—fatty acids; OS—other substances.

**Table 4:** Only cannabinoids content in the inflorescences (H_IF) and leaves (H_L) of hemp in a chloroform extract mixture obtained by the Bligh and Dyer method.

Item		H_IF	H_L	H_IF	H_L
		% *		%/100 g DM	
		Mean (±SD)		Mean (±SD)	
2-Norpinanol 3,6,6-trimethyl	C_10_H_18_O	1.55 ^a^ (±0.03)	0.48 ^b^ (±0.00)	0.20 ^a^ (±0.00)	0.03 ^b^ (±0.00)
Phytyl, 2-methylbuanoate	C_25_H_48_O_2_	2.92 ^a^ (±0.03)	0.65 ^b^ (±0.03)	0.37 ^a^ (±0.01)	0.05 ^b^ (±0.00)
Phytol	C_20_H_40_O	3.90 ^a^ (±0.07)	9.55 ^b^ (±0.21)	0.49 ^a^ (±0.01)	0.70 ^b^ (±0.01)
Hexacosane	C_26_H_55_	10.33 ^a^ (±0.11)	12.21 ^b^ (±0.23)	1.30 ^a^ (±0.03)	0.90 ^b^ (±0.04)
Octacosane	C_28_H_30_O_2_	1.19 ^a^ (±0.00)	1.69 ^b^ (±0.02)	0.15 ^a^ (±0.00)	0.12 ^b^ (±0.00)
Cannabidiol	C_21_H_30_O_2_	2.03 ^a^ (±0.05)	0.00 ^b^ (±0.00)	0.26 ^a^ (±0.01)	0.00 ^b^ (±0.00)
Cannabichromene	C_21_H_30_O_2_	5.78 ^a^ (±0.25)	0.00 ^b^ (±0.00)	0.73 ^a^ (±0.04)	0.00 ^b^ (±0.00)
Δ8-Tetrahydrocannabinol	C_21_H_30_O_2_	7.83 ^a^ (±0.04)	0.00 ^b^ (±0.00)	0.99 ^a^ (±0.01)	0.00 ^b^ (±0.00)
Hexacosanoic acid, methyl ester	C_27_H_54_O_2_	0.86 ^a^ (±0.03)	0.34 ^b^ (±0.04)	0.11 ^a^ (±0.00)	0.02 ^b^ (±0.00)

* The percentage content in total fatty acids and other fatty substances determined. a, b—Values in the same row with different superscript letters are significantly different at *p* ≤ 0.05. SD—Standard deviation (*n* = 3).

**Table 5:** Values of lipid quality indices in the inflorescences (H_IF) and leaves (H_L) of hemp.

	H_IF	H_L
Index	Mean (±SD)	
AI	0.39 ^a^ (±0.01)	0.58 ^b^ (±0.01)
AI (+C18:0)	3.02 ^a^ (±0.09)	4.66 ^b^ (±0.11)
TI	0.17 ^a^ (±0.00)	0.24 ^b^ (±0.00)
h/H	2.94 ^a^ (±0.05)	1.91 ^b^ (±0.05)
HPI	2.58 ^a^ (±0.05)	1.73 ^b^ (±0.04)

AI—atherogenic index; TI—thrombogenic index; h/H—hypocholesterolemic/hypercholesterolemic ratio; HPI—health-promoting index. a, b—Values in the same row with different superscript letters are significantly different at *p* ≤ 0.05. SD—Standard deviation (*n* = 3).

**Table 6:** Overview of lipid quality indices used.

Index	Equation	
Atherogenic Index (AI)	[C12:0 + (4 × C14:0) + C16:0]/ΣUFA	(2)
Corrected Atherogenic Index (AI)	[C12:0 + (4 × C14:0) + C16:0]/ΣUFA + C18:0	(3)
Thrombogenic Index (TI)	(C14:0 + C16:0 + C18:0)/((0.5 × C18:1) + (0.5 × other MUFA) + (0.5 × Σn-6 PUFA) + (3 × Σn-3 PUFA) + Σn-3 PUFA/Σn-6 PUFA)	(4)
Hypocholesterolemic/Hypercholesterolemic Ratio (h/H)	(C18:1 + ΣPUFA)/(C12:0 + C14:0 + C16:0)	(5)
Health-Promoting Index (HPI)	ΣUFA/[C12:0 + (4 × C14:0) + C16:0]	(6)

## Data Availability

The original contributions presented in this study are included in the article. Further inquiries can be directed to the corresponding author(s).

## Supplementary Materials

The following supporting information can be downloaded at: https://www.mdpi.com/article/10.3390/molecules30091947/s1, Table S1: Descriptive statistics for hemp inflorescences (H_IF) (*n* = 3) results; Table S2: Descriptive statistics for hemp leaves (H_L) (*n* = 3) results; Figure S1: Mass spectrum for Δ8-THC from experimental and library spectra; Figure S2: Mass spectrum for cannabidiol from experimental and library spectra.

## Abbreviations

The following abbreviations are used in this manuscript:

THCA	Tetrahydrocannabinolic acid
CBDA	Cannabidiolic acid
CBGA	Cannabigerolic acid
CBD	Cannabidiol
PUFAs	Polyunsaturated fatty acids
MUFAs	Monounsaturated fatty acids
SFAs	Saturated fatty acids
AI	Atherogenic index
TI	Thrombogenic index
HPI	Health-promoting index
h/H	Hypocholesterolemic/hypercholesterolemic fatty acid ratio
H_IF	Hemp inflorescences
H_L	Hemp leaves
DM	Dry matter
CF	Crude fat
SD	Standard deviation
UFAs	Unsaturated fatty acids
FAs	Fatty acids
OS	Other substances
CBC	Cannabichromene
Δ8-THC	Δ8-Tetrahydrocannabinol
Δ9-THC	Δ9-Tetrahydrocannabinol
FAMEs	Fatty acid methyl esters
GCMS	Gas chromatography mass spectrometry
tR	Retention time
DFAs	Hypocholesterolemic fatty acids
OFAs	Hypercholesterolemic fatty acids
