# Supplementary material for: Fatty Acid Composition and Bioactive Profiles in the Aerial Parts of Cannabis sativa
Source: Molecules. 2025 Apr 27;30(9):1947. doi: 10.3390/molecules30091947 (PMC12073161; doi:10.3390/molecules30091947)
Supplement: Supplementary file 1 [file molecules-30-01947-s001.zip › molecules-3553055-supplementary.pdf]

molecules

MDPI

Table S1. Descriptive statistics for hemp inflorescences (H_IF) (n = 3) results.

Variables (n = 3)	Mean	Median	Minimum	Maximum	Lower quartile	Upper quartile	Standard deviation
Dry matter	89.67	89.67	89.62	89.73	89.62	89.73	0.05
Crude fat	12.63	12.55	12.54	12.79	12.54	12.79	0.14
C12:0	0.27	0.27	0.27	0.28	0.27	0.28	0.00
C14:0	0.63	0.64	0.60	0.66	0.60	0.66	0.03
C16:0	11.6	11.74	11.24	11.82	11.24	11.82	0.31
C17:0	0.74	0.73	0.73	0.77	0.73	0.77	0.02
C18:0	2.63	2.65	2.54	2.70	2.54	2.70	0.08
C20:0	2.53	2.51	2.48	2.59	2.48	2.59	0.06
C22:0	4.80	4.78	4.41	5.20	4.41	5.20	0.40
C24:0	3.27	3.18	3.14	3.49	3.14	3.49	0.19
C16:1 n–7	0.39	0.38	0.38	0.40	0.38	0.40	0.01
C18:1 n–9	2.23	2.23	2.22	2.25	2.22	2.25	0.02
C18:2 n–6	8.13	8.10	8.07	8.21	8.07	8.21	0.07
C18:3 n–3	26.39	26.31	25.90	26.96	25.90	26.96	0.53
2-Norpinanol 3,6,6-trimethyl	1.55	1.54	1.53	1.58	1.53	1.58	0.03
Phytyl, 2-methylbuanoate	2.92	2.91	2.90	2.96	2.90	2.96	0.03
Phytol	3.90	3.87	3.86	3.98	3.86	3.98	0.07
Hexacosane	10.33	10.27	10.26	10.46	10.26	10.46	0.11
Octacosane	1.19	1.19	1.19	1.19	1.19	1.19	0.00
Cannabidiol	2.03	2.03	1.97	2.08	1.97	2.08	0.05
Cannabichromene	5.78	5.82	5.51	6.01	5.51	6.01	0.25
Δ8-tetrahydrocannabinol	7.83	7.84	7.78	7.86	7.78	7.86	0.04
Hexacosanoic acid, methyl ester	0.86	0.85	0.83	0.89	0.83	0.89	0.03
SFA	26.48	26.57	26.23	26.63	26.23	26.63	0.21
MUFA	2.62	2.62	2.60	2.63	2.60	2.63	0.02
PUFA	34.52	34.52	33.98	35.06	33.98	35.06	0.54
UFA	37.14	37.15	36.61	37.66	36.61	37.66	0.53
n–3	26.39	26.31	25.90	26.96	25.90	26.96	0.53
n–6	8.13	8.10	8.07	8.21	8.07	8.21	0.07
n–6/n–3	0.31	0.31	0.30	0.31	0.30	0.31	0.01
AI	0.39	0.39	0.38	0.40	0.38	0.40	0.01
AI (with C18:0)	3.02	3.04	2.92	3.09	2.92	3.09	0.09
TI	0.17	0.17	0.17	0.17	0.17	0.17	0.00
h/H	2.94	2.95	2.88	2.99	2.88	2.99	0.05
HPI	2.58	2.59	2.52	2.63	2.52	2.63	0.05

molecules

MDPI

Table S2. Descriptive statistics for hemp leaves (H_L) (*n* = 3) results.

Variables (*n* = 3)	Mean	Median	Minimum	Maximum	Lower quartile	Upper quartile	Standard deviation
Dry matter	89.11	89.11	89.08	89.14	89.08	89.14	0.03
Crude fat	7.35	7.38	7.12	7.55	7.12	7.55	0.22
C12:0	0.31	0.31	0.31	0.32	0.31	0.32	0.01
C14:0	0.92	0.93	0.91	0.93	0.91	0.93	0.01
C16:0	18.95	18.74	18.67	19.44	18.67	19.44	0.42
C17:0	0.23	0.23	0.23	0.23	0.23	0.23	0.00
C18:0	4.08	4.11	3.94	4.18	3.94	4.18	0.12
C20:0	4.03	3.96	3.85	4.27	3.85	4.27	0.21
C22:0	3.79	3.79	3.76	3.82	3.76	3.82	0.03
C24:0	3.00	3.10	2.80	3.11	2.80	3.11	0.18
C16:1 n–7	1.29	1.30	1.28	1.30	1.28	1.3	0.01
C18:1 n–9	1.99	1.96	1.90	2.11	1.90	2.11	0.11
C18:2 n–6	7.00	7.02	6.78	7.21	6.78	7.21	0.22
C18:3 n–3	29.48	29.51	29.32	29.63	29.32	29.63	0.16
2-Norpinanol 3,6,6-trimethyl	0.47	0.48	0.47	0.48	0.47	0.48	0.00
Phytyl, 2-methylbuanoate	0.65	0.65	0.62	0.69	0.62	0.69	0.03
Phytol	9.55	9.45	9.41	9.78	9.41	9.78	0.21
Hexacosane	12.21	12.32	11.94	12.36	11.94	12.36	0.23
Octacosane	1.69	1.69	1.68	1.71	1.68	1.71	0.02
Cannabidiol	0.00	0.00	0.00	0.00	0.00	0.00	0.00
Cannabichromene	0.00	0.00	0.00	0.00	0.00	0.00	0.00
Δ8-tetrahydrocannabinol	0.00	0.00	0.00	0.00	0.00	0.00	0.00
Hexacosanoic acid, methyl ester	0.34	0.32	0.32	0.39	0.32	0.39	0.04
SFA	35.32	35.19	35.16	35.59	35.16	35.59	0.24
MUFA	3.28	3.26	3.20	3.39	3.20	3.39	0.10
PUFA	36.49	36.52	36.28	36.65	36.28	36.65	0.18
UFA	39.77	39.91	39.49	39.92	39.49	39.92	0.25
n3	29.48	29.51	29.32	29.63	29.32	29.63	0.16
n6	7.00	7.02	6.78	7.21	6.78	7.21	0.22
n6/n3	0.24	0.24	0.23	0.25	0.23	0.25	0.01
AI	0.58	0.57	0.57	0.59	0.57	0.59	0.01
AI (with C18:0)	4.66	4.68	4.54	4.75	4.54	4.75	0.11
TI	0.24	0.24	0.24	0.25	0.24	0.25	0.00
h/H	1.91	1.93	1.85	1.94	1.85	1.94	0.05
HPI	1.73	1.75	1.68	1.76	1.68	1.76	0.04

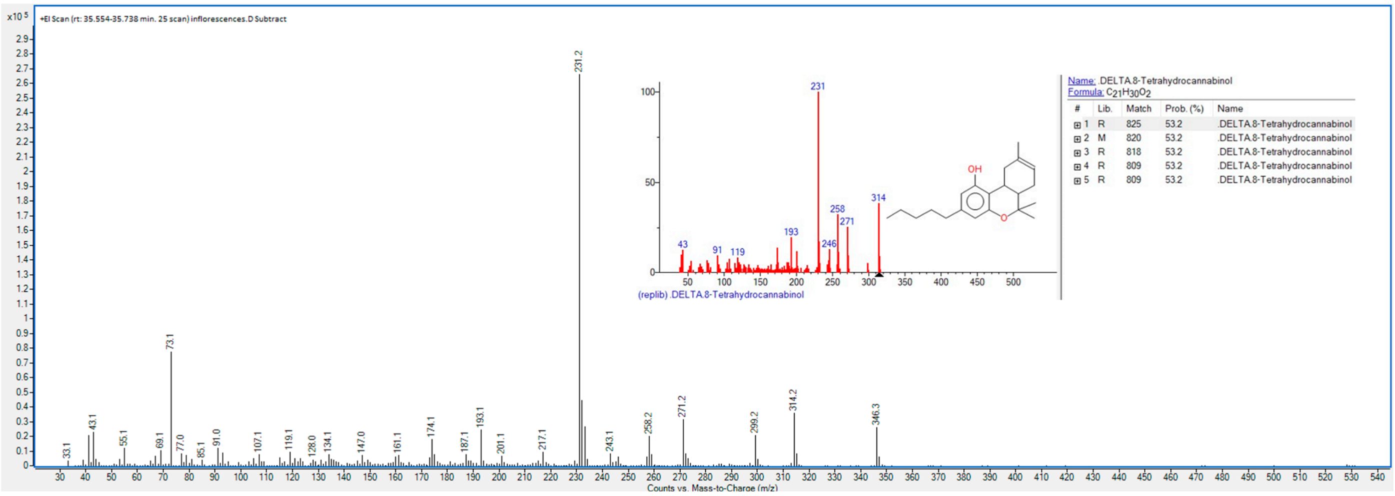

Figure S1. Mass spectrum for Δ8-THC from experimental and library spectra.

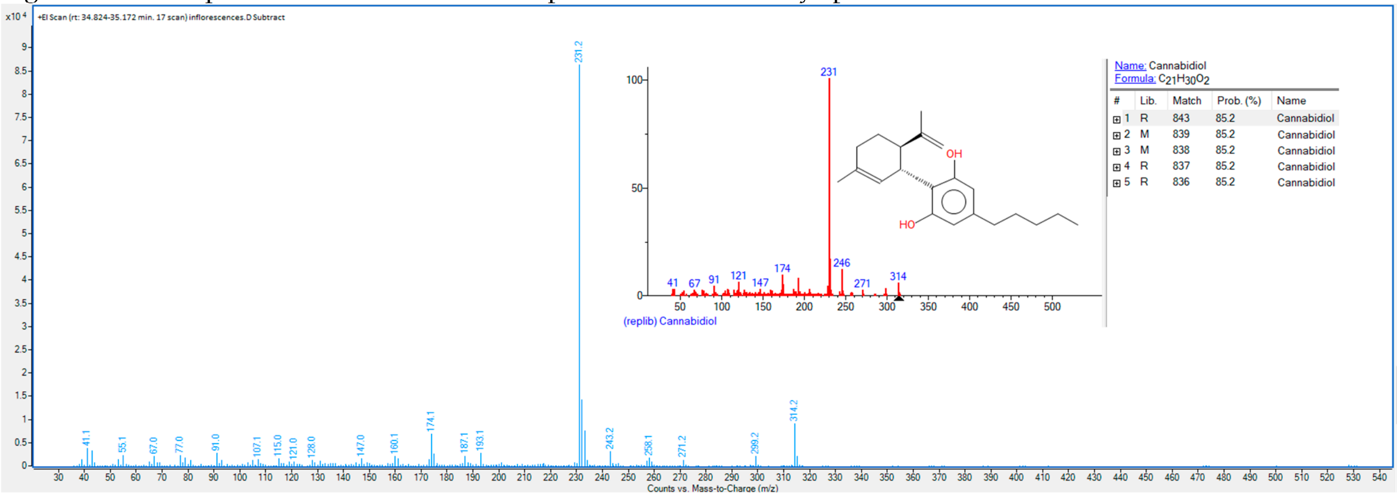

Figure S2. Mass spectrum for Cannabidiol from experimental and library spectra.
